# Evaluating the effect of a proposed logistics fee cap on pharmaceuticals in South Africa - a pre and post analysis

**DOI:** 10.1186/s12913-015-1184-6

**Published:** 2015-11-26

**Authors:** Varsha Bangalee, Fatima Suleman

**Affiliations:** Institution: Discipline of Pharmaceutical Sciences, School of Health Sciences, Westville Campus, University of KwaZulu-Natal, Private Bag X54001, Durban, 4000 South Africa

**Keywords:** Logistics fees, Medicine pricing, Price caps, Single exit pricing

## Abstract

**Background:**

South Africa has proposed the implementation of a maximum logistics fee paid by pharmaceutical manufacturers to wholesalers and distributors. However very little knowledge exists of the effects, unintended or otherwise, of the implementation of these proposed regulations, which are required to guide further policy development and implementation. The objectives of this study was to therefore evaluate the effects of the proposed logistics fee cap on different pharmaceuticals and different dosage forms, as well as to observe the logistics fee contribution to the Single Exit Price.

**Methods:**

Private sector medicine prices were sourced from the South African Medicine Price Registry as at 20 December 2013. For each medicine the maximum logistics fee was calculated based on the 2012 proposed government guidelines. The logistics fee as a percentage of the final Single Exit Price was calculated, as part of the analysis of results.

**Results:**

Out of the 47 medicines in the overall sample from the current study, only 16 medicines showed a decrease in the Single Exit Price with the application of the maximum logistics fee cap.

**Conclusion:**

This study reveals the need for greater transparency of the mark ups along the distribution chain as well as further research with regards to the costing of logistics fees of similar pharmaceuticals.

## Background

South Africa is classified as a middle-income country with a per capita total expenditure on health (PPP int. $) of 843 and a population of 47.3 million [1]. According to the Organisation for Economic Co-operation and Development (OECD) report (2011), approximately 8.5 % of GDP was spent on health care, which was above the 5 % recommended by the World Health Organisation (WHO) and relatively high by international standards [2]. Healthcare delivery in South Africa is characterised by a significant private sector operating alongside a public health sector. The public health sector which is largely tax funded, remains under pressure to deliver services to nearly 85 % of the population [3]. Approximately 16 % of the population have access to medical insurance, while the rest of the population accessing the public sector, also make out-of-pocket purchases in the private sector. The bulk of private funding comes from medical aid contributions (66 %) and out-of-pocket payments (29.7 %) [2].

In 1994, South Africa had seen the demise of apartheid, a dysfunctional regime which had resulted in a healthcare system grappling with a high disease burden and facing several economic challenges, many of which still persist today [4]. The high price of medicines in a previously largely unregulated medicines market posed one of the primary challenges. Cameron et al., in their research on medicine prices in 36 countries, have stated that the structure of a country’s pharmaceutical sector can influence medicine availability, price, and affordability. They recommended that further research be conducted to establish appropriate mark-up levels that promote medicine availability and affordability while ensuring that the supply chain remained viable [5]. Brazil has also experienced a combination of poor distribution of essential medicines by the public sector and weak legislation on medicine pricing that impacted on access to medicines and placed additional financial burdens on patients. Price composition data supplied by pharmaceutical companies to Brazil’s Parliament suggested that the cost of production made up 42.6 % of the retail price of a medicine. The Productivity Commission study on international medicine prices, carried out in Australia found that manufacturers’ prices for a given basket of 150 medicines in Australia was nearly 50 % more in Sweden and 200–300 % higher, on average, in the USA [6]. Financial accessibility was one of the barriers identified by Bigdeli et al. in terms of access to medicines [7].

South Africa’s response to the pricing issue was for the government to initiate a policy review which led to the development of the National Drugs Policy for South Africa (NDP) to address the health, economic and national development objectives for the country. The policy document called for the establishment of a Pricing Committee which would be committed to “total transparency in the pricing structure of pharmaceutical manufacturers, wholesalers, providers of services, such as dispensers of drugs, as well as private clinics and hospitals” [8].

Improving access to medicines in South Africa centres on the establishment of a transparent and institutionally strong supply chain. The prices of medicines themselves are affected by the manufacturer’s selling prices, duties, taxes and mark-ups along the supply chain [9]. The South African pharmaceutical supply chain consists of a complex web of heterogeneous stakeholders functioning amidst a highly competitive market [10]. The past decade has seen several industry and regulatory changes which were instituted to increase accessibility of medicines to the South African population [11]. Policies and regulatory changes were aimed to reduce the prices of medicines and control the mark-up instituted by the various stakeholders along the entire supply chain from the manufacturer to the final medicine dispenser.

The earliest of these regulations, was the institution of a Single Exit Price (SEP) system in August 2004 [12]. The SEP, which consists of the ex-manufacturer’s price, logistics fee and value added tax (VAT), also represents the selling price to wholesalers. Prior to the inception of SEP, logistics fees were added to the wholesaler selling price [13]. With the implementation of the SEP, the logistics fee which covers the cost of distribution of pharmaceuticals is negotiated privately between the importer or manufacturer and the logistics service provider. The logistics fee is generally between 10 and 15 % of the final price [9]. The process is not made transparent however, and the final logistics fee in the Databases of Medicine Prices is as reflected in the SEP information provided to the National Department of Health by manufacturers. This however may not be a true reflection of negotiated agreements applied in practice for logistics fees, and any additional savings on the logistics fee portion of the SEP accrues back to the manufacturer (which effectively means a higher ex-manufacturer price).

On recommendations from the South African Pricing Committee, charged with ensuring a transparent pricing structure, the introduction of a maximum logistics fee paid by pharmaceutical manufacturers to wholesalers and distributors to get their medicines to pharmacies and doctors has been proposed. The draft regulation which has yet to be implemented was published in the Government Gazette*,* on 18^th^ September 2012, and has set forth four price bands with different maximum logistics fees for each band subject to the ex-manufacturer price [14]. Since its publication in the Gazette, there has been much debate amongst industry players, particularly from smaller firms who argue that the proposed regulation will allow larger pharmaceutical companies to negotiate logistics fees below the maximum set by the government [15]. Further industry concerns exist as to whether stakeholders will be assured appropriate remuneration for their services [16]. The comment period would have ended on the 18^th^ of December 2012, and had the proposed logistics fee be implemented, 2013 would have been the first year of implementation, but this process has been delayed due to further negotiations between Government and the various stakeholders.

The high health expenditure in developing and transitional countries (20–60 % as compared with 18 % in countries of the Organisation for Economic Co-operation and Development) [5] has long been documented. The accessibility and availability of medicines in these countries is also synonymously well known. In several of these countries, the pharmaceutical supply chain is neither regulated nor subject to any formal oversight and, as a result contributes to problems of availability and affordability of medicines [9]. Despite some interventions in the supply chain being implemented, there is a general dearth of evidence in the literature pertaining to the implementation and outcomes of pricing regulations in low- and middle-income countries, especially in terms of fees along the supply chain. This probably results from interventions being implemented by government agencies without proper monitoring of effects of policies, data not being systematically collected or not usually reaching publication status. This therefore warrants the need for more research into South Africa’s attempt to manage supply chain prices, so as to provide other countries with lessons learnt from this process.

This prospective analysis will therefore provide insight into proposed logistics fee caps, in an effort to guide further regulation recommendations. This could importantly serve as a platform and add to the current body of evidence on pricing interventions in the pharmaceutical supply chain in Low and Middle Income countries and in turn, present evidence on the complexity of the pharmaceutical policy development and implementation. It will further aid interested parties in identifying the characteristics, strengths and limitations of the proposed regulation with the aim of facilitating informed pharmaceutical policy decision-making in general to improve the access and affordability of medicines.

The primary objective of this paper is to examine the pre and post effects of the 2013 proposed logistics fees regulation on the price of originator and generic medicines of several subgroups of pharmaceutical dosage forms. A secondary objective is to observe the total contribution of the logistics fee toward the final SEP.

## Methods

### Data source

The empirical analysis used data retrieved from the South African Medicine Price Registry on private sectors prices for originator and generic medicines as at 20 December 2013. The dataset was chosen as it represents the year in which the proposed logistics fee cap was likely to have been implemented post the draft Gazette publication in 2012. The December 2013 dataset was selected as all price changes (SEP adjustments) for the year would have been completed and a comparative analysis could thus be made.

### Sample selection

The sample of non-cold chain medicines was derived from the World Health Organisation (WHO)/Health Action International (HAI) methodology global core list [17], with insulin preparations available for the treatment of type 1 diabetes in South Africa representing the remaining cold chain sample. The global core list comprises of 14 medicines that are included in all medicine price surveys, i.e. salbutamol, glibenclamide, atenolol, captopril, simvastatin, amitriptyline, ciprofloxacin, co-trimoxazole , amoxicillin, ceftriaxone, diazepam, diclofenac, paracetamol, and omeprazole. These medicines were used as the basis to identify all other dosage forms of the same product. As this study aimed to compare the effect of the proposed price cap on both originator and generic medicines, only generic price data for which data was also available for the originator medicine were included, hence the exclusion of glibenclamide, amitriptyline and co-trimoxazole from the original global core list. An originator medicine was compared with the price of a generic medicine if both medicines had the same active substance, same pharmaceutical dosage form and same strength. All data for medicines that were discontinued were omitted from the analysis. The pricing data for paracetamol tablets and syrup (both schedule zero) were not included as schedule zero medications as well as veterinary medicines are currently exempt from the pricing regulations in South Africa.

### Data analysis

Based on the selected sample list, the price data for all available dosage forms of the selected medicines were collected and expressed in South African Rand (ZAR). The SEP is made up of the ex-manufacturers price, logistics fee and VAT. VAT is charged at a standard 14 % across the board and is calculated from the sum of the ex-manufacturers price and the logistics fee and added to the combination of these 2 prices.

#### Pricing pre-logistics fee regulation

The ex-manufacturer price, logistics fee and single exit unit price inclusive of VAT (to ensure comparability of medicine prices owing to differences in pack sizes between manufacturers) for the originator and the lowest priced generic for each sample medicine as at 20 December 2013 was recorded.

#### Pricing post-logistics fee regulation

For each medicine the maximum logistics fee was calculated based on the 2012 proposed government guidelines (Prices were based on the ex-manufacturer price excluding VAT):

- For medicines where the ex-manufacturer price is ZAR 100 or less (excluding VAT) the fee can be no more than 8 % plus ZAR 3.
- For medicines priced between ZAR 100 and ZAR 500, the fee is capped at 6 % plus ZAR 4.
- For medicines priced at ZAR 500 and above but less than ZAR1 000 the fee is capped at 4 % plus ZAR 5.
- Medicines priced at ZAR 1 000 or higher will have logistics fees capped at ZAR 54.

The logistics fee as a percentage of the final Single Exit Price was calculated, as part of the analysis of results. The median logistics fee as a percentage of the SEP was calculated for the originator and lowest priced generics for all dosage forms surveyed. The results reported in the article, reflect a segment of a lengthier survey, available upon request. The proposed regulation was statistically evaluated using a paired *t*-test to determine whether the paired observations are significantly different from one another for both originator and generic medicines respectively for all dosage forms sampled. A confidence interval of 95 % was considered significant.

Ethical approval was obtained from the University Of KwaZulu-Natal Humanities and Social Science Research Ethics Committee (HSS/0154/013).

## Results

The empirical analysis of the proposed logistics fee is reported below, along with estimated parameters for models of both originator and generic medicines for 4 different dosage forms, with the inclusion of a cold chain dataset. In addition to exploiting the before–after policy variation in prices, we also make comparisons between the percentage logistics fees of the various dosage forms.

Tables 1, 2, 3 and 4 reflect the ex-manufacturers price, logistics fees, unit price and SEP for oral solid, oral liquid, injectable dosage forms and insulin cold chain products. These tables demonstrate the effects of applying the proposed maximum logistics fee cap to each medicine and dosage form in the dataset compared to the current policy.

**Table 1:** Solid oral dosage forms

				Pre-Logistics Fee Regulation Prices				Post-Logistics Fee Regulation Prices					
Active Ingredients	O/G	Pack Size	Ex-Manufacturers Price in 2013	Logistics Fee in 2013	SEP in 2013 (VAT incl.)	Unit Price in 2013	Logistics fee/SEP %	Logistics fee with proposed cap	SEP with proposed cap (VAT incl.)	Unit Price with proposed cap	Logistics fee with proposed cap/Sep %	Change in SEP after logistics fee cap	Overall effect on SEP after logistics fee cap
Omeprazole 20 mg tab	O	28	426.33	14.92	503.03	17.97	2.97	29.58	519.74	18.57	5.7	16.71	Increase
Omeprazole 20 mg cap	G	28	86.99	15.35	116.66	4.17	13.16	9.96	119.08	4.25	8.36	2.42	Increase
Amoxicillin Trihydrate 500 mg cap	O	100	474.40	71.16	621.94	6.22	11.45	32.46	577.83	5.78	5.62	−44.11	Decrease
Amoxicillin Trihydrate 500 mg cap	G	100	60.71	5.16	75.09	0.75	6.88	7.86	78.17	0.79	10.06	3.08	Increase
Atenolol 50 mg tab	O	30	156.57	5.48	184.74	6.16	2.97	13.39	193.76	6.46	6.92	9.02	Increase
Atenolol 50 mg tab	G	30	12.19	1.21	15.28	0.51	7.92	3.98	18.43	0.62	21.58	3.15	Increase
Captopril 25 mg tab	O	60	128.08	20.53	169.42	2.83	12.12	11.68	159.33	2.66	7.34	−10.09	Decrease
Captopril 25 mg tab	G	60	11.86	0.71	14.33	0.24	4.96	3.95	18.02	0.31	21.92	3.69	Increase
Simvastatin 20 mg tab	O	28	57.23	6.53	72.68	2.6	8.99	7.58	73.88	2.64	10.26	1.2	Increase
Simvastatin 20 mg tab	G	28	16.97	1.7	21.28	0.77	7.98	4.36	24.31	0.87	17.93	3.03	Increase
Ciprofloxacin 500 mg tab	O	10	115.97	10.68	144.38	14.44	7.4	10.96	144.69	14.47	7.58	0.31	Increase
Ciprofloxacin 500 mg tab	G	10	8.75	1.25	11.4	1.21	10.97	3.7	14.19	1.42	26.07	2.79	Increase
Diclofenac Sodium 50 mg tab	O	20	36.67	3.58	45.88	2.3	7.8	5.93	48.57	2.43	12.22	2.69	Increase
Diclofenac Sodium 50 mg tab	G	20	9.33	1.39	12.23	0.61	11.37	3.75	18.13	0.91	20.68	5.9	Increase

Notes: All Prices are in ZAR; O = Originator brand G = Generic brand; Originator Solid oral dosage forms: *P* value = 0.658; Generic solid oral dosage forms: *P* value = 0.0002

**Table 2:** Injectable dosage forms

				Pre-Logistics Fee Regulation Prices				Post-Logistics Fee Regulation Prices					
Active Ingredients	O/G	Pack Size	Ex-Manufacturers Price in 2013	Logistics Fee in 2013	SEP in 2013 (VAT incl.)	Unit Price in 2013	Logistics fee/SEP %	Logistics fee with proposed cap	SEP with proposed cap (VAT incl.)	Unit Price with proposed cap	Logistics fee with proposed cap/Sep %	Change in SEP after logistics fee cap	Overall effect on SEP after logistics fee cap
Omeprazole 40 mg/10 ml	O	100 ml	219.09	0.66	250.51	2.51	0.26	17.15	269.31	2.69	6.37	18.79	Increase
Omeprazole 40 mg/10 ml	G	10 ml	67.77	11.96	90.89	9.09	13.16	8.42	86.85	8.69	9.69	−4.03	Decrease
Diazepam 10 mg/2 ml	O	10 ml	101.43	10.62	127.73	12.77	8.313	10.1	127.12	12.71	7.93	−0.61	Decrease
Diazepam 10 mg/2 ml	G	20 ml	23.36	1.40	28.23	1.41	4.97	4.87	32.18	1.61	15.13	3.95	Increase
Ciprofloxacin 2 mg/ml	O	50 ml	74.53	6.63	92.52	1.85	7.17	8.96	95.18	1.90	9.42	2.66	Increase
Ciprofloxacin 2 mg/ml	G	50 ml	23.71	1.92	29.22	0.58	6.58	4.89	32.61	0.65	15.02	3.39	Increase
Diclofenac 75 mg/3 ml	O	15 ml	43.81	4.28	54.82	3.65	7.79	6.51	57.36	3.82	11.34	2.54	Increase
Diclofenac 75 mg/3 ml	G	150 ml	43.66	4.85	55.31	0.37	8.77	6.49	57.18	0.38	11.36	1.87	Increase
Paracetamol 500 mg/50 ml	O	100 ml	283.75	45.68	371.99	0.31	11.44	21.03	347.45	3.52	3.60	−24.54	Decrease
Salbutamol 500mcg/ml	O	5 ml	156.06	27.53	209.29	41.86	13.16	13.36	193.14	193.14	6.92	−16.15	Decreased
Ceftriaxone 1 g	O	3.5 mL	57.88	6.063	72.89	20.83	8.32	7.63	74.68	21.34	10.22	1.79	Increase
Ceftriaxone 1 g	G	3.5 mL	9.522	0.94	11.93	3.41	7.89	3.76	15.14	4.33	24.84	3.21	Increase

All Prices are in ZAR; O = Originator brand G = Generic brand; Originator injectable dosage forms: *P* value = 0.693; Generic injectable dosage forms: *P* value = 0.318

**Table 3:** Oral liquid dosage forms

				Pre-Logistics Fee Regulation Prices				Post-Logistics Fee Regulation Prices					
Active Ingredients	O/G	Pack Size	Ex-Manufacturers Price in 2013	Logistics Fee in 2013	SEP in 2013 (VAT incl.)	Unit Price in 2013	Logistics fee/SEP %	Logistics fee with proposed cap	SEP with proposed cap (VAT incl.)	Unit Price with proposed cap	Logistics fee with proposed cap/Sep %	Change in SEP after logistics fee cap	Overall effect on SEP after logistics fee cap
Salbutamol 2 mg/5 ml syr.	O	200 ml	45.15	1.58	53.28	0.27	2.97	6.61	59.01	0.29	11.20	5.74	Increase
Salbutamol 2 mg/5 ml syr.	G	100 ml	7.91	0.88	10.02	0.1	8.78	3.63	13.16	0.13	27.61	3.14	Increase
Ciprofloxacin 250 mg/5 ml sus	O	100 ml	158.92	14.03	197.16	1.97	7.12	13.54	196.59	1.97	6.88	−0.56	Decrease

All Prices are in ZAR; O = Originator brand G = Generic brand; Originator oral liquid dosage forms: *P* value = 0.56; Generic oral liquid dosage forms: *P* value = could not be obtained as only one set of variables

**Table 4:** Insulin preparations (Cold-chain)

				Pre-Logistics Fee Regulation Prices				Post-Logistics Fee Regulation Prices					
Active Ingredients	O/G	Pack Size & Quantity	Ex- Man.Price in 2013	Log Fee in 21013	SEP in 2013 (VAT incl.)	Unit Price in 2013	Logistics fee/SEP %	Logistics fee with proposed cap	SEP with proposed cap (VAT incl.)	Unit Price with proposed cap	Logistics fee with proposed cap/Sep %	Change in SEP after logistics fee cap	Overall effect on SEP after logistics fee cap
Ultra-fast acting													
Insulin Glulisine	O	10/1	258.79	8	304.14	30.41	2.63	19.53	317.28	31.73	6.15	13.14	Increase
Insulin aspart	O	10/1	223.63	30.19	289.36	28.94	10.44	17.42	274.79	27.48	6.34	−14.57	Decrease
Insulin Lispro	O	10/1	210.08	12.60	253.86	25.39	4.97	16.60	258.42	25.84	6.43	4.5694	Increase
Fast acting													
Insulin (Dna)	O	10/1	193.64	26.14	250.56	25.06	10.43	15.62	238.56	23.86	6.55	−11.99	Decrease
Human Insulin	G	5/1	240.25	19.22	295.79	59.16	6.49	18.41	294.87	58.97	6.244	−0.92	Decrease
Regular Human Insulin (Rdna)	O	10/1	210.08	12.60	253.86	25.39	4.97	16.60	258.42	25.84	6.43	4.56	Increase
Intermediate to long acting													
Human Insulin	G	5/1	240.25	19.22	295.79	59.16	6.49	18.41	294.87	58.97	6.244	−0.92	Decrease
Isophane insulin	O	10.1	233.46	31.52	302.07	30.21	10.43	18.01	286.67	28.67	6.28	−15.40	Decrease
Isophane Human Insulin (Rdna)	O	10/1	237.81	14.27	287.37	28.74	4.97	18.27	291.93	29.19	6.26	4.56	Increase
Long acting Insulin analogues													
Insulin Glargine	O	3/5	505.68	23.83	603.64	40.24	3.95	25.23	605.23	40.35	4.17	1.59	Increase
Insulin Detemir	O	3/5	460.73	62.19	596.14	39.74	10.43	31.64	561.30	37.42	5.64	−34.83	Decrease
Biphasic													
Biosynthetic human insulin	O	3/5	458.27	61.87	592.96	39.53	10.44	31.49	558.34	37.22	5.64	−34.61	Decrease
Biosynthetic Human Insulin (30 % Regular Insulin and 70 % Isophane Insulin)	O	3/5	349.13	22.29	423.41	28.23	5.26	24.95	426.45	28.43	5.85	3.04	Increase
Insulin (Human)	G	3/5	321.71	15.28	384.17	25.61	3.98	23.30	393.31	26.22	5.92	9.14	Increase
Human Insulin/ isophane insulin	G	5/1	235.21	18.82	289.59	57.92	6.49	18.11	288.79	57.76	6.27	−0.80	Decrease
Biphasic Insulin analogues													
75 % Insulin Lispro Protamine Suspension (Npl)	O	3/5	349.12	22.28	423.39	28.23	5.26	24.95	426.43	28.43	5.85	3.04	Increase
Insulin aspart	O	3/5	417.34	56.34	539.99	35.99	10.43	29.040	508.87	33.92	5.71	−31.12	Decrease
50 % Insulin Lispro Protamine Suspension (Npl)	O	3/5	349.12	22.28	423.39	28.23	5.26	24.95	426.43	28.43	5.85	3.04	Increase

All Prices are in ZAR; O = Originator brand; G = Generic brand; Originator insulin preparations: *P* value = 0.148; Generic insulin preparations: *P* value = 0.808

### Solid oral dosage forms

The sample size for solid oral dosage forms consisted of seven representatives. With the exception of the logistics fee for omeprazole tablets, six out of the seven solid oral dosage forms that were analysed indicated that the ex-manufacturer price, the logistics fee and the final SEP for the originator medicines were higher than that of the generic medicines (see Table 1). An expression of the logistics fee as a percentage of the SEP however reveals that four out of the seven generic medicines, namely omeprazole capsules, atenolol, ciprofloxacin and diclofenac tablets had a higher percentage of the logistics fee make-up of the final SEP.

With the application of the maximum proposed logistics fees, the logistics fees for the generic medicines remained less than that of the originator medicines; however the expression of the logistics fee as a percentage of the SEP was higher for all generic medicines. The SEP for all medicines (originator and generic) in the data set had increased with the exception of originator amoxicillin capsules and captopril tablets. This reveals that for only two of the analysed medicines, the logistics fees percentages were above the proposed cap, hence the cap will reduce these prices.

### Injectable dosage forms

From the global core WHO/HAI list, ceftriaxone, omeprazole, diazepam, ciprofloxacin, diclofenac, paracetamol and salbutamol were available as injectable dosage forms. Paracetamol and salbutamol currently do not have any registered generic injectable medicines in South Africa. Similar to the oral dosage form, for all of the injectable dosage forms, the ex-manufacturer price, logistics fee and the final SEP for the originator medicines were higher than that of the generic medicines, with two exceptions (see Table 2). The logistics fees as well as the percentage logistics fee of the SEP for generic omeprazole and diclofenac were higher than those of the originator.

The application of the maximum proposed logistics fee has produced an overall increase in the single exit price for all originator and generic pairings with the exception of the originator diazepam and the generic omeprazole injections. Interestingly it does however result in a decreased SEP for paracetamol and salbutamol injections for which there were no generics available.

### Oral liquid dosage forms

Salbutamol syrup and ciprofloxacin suspension represented the two liquid oral preparations analysed in this study (see Table 3). Currently there are no generic preparations available for ciprofloxacin in South Africa. Despite originator salbutamol syrup having a higher ex-manufacturer price, logistics fee and SEP unit price, the generic preparation had a higher logistics fee component of the final SEP.

The application of the maximum proposed logistics fee had resulted in an overall final price increase for both generic and originator brands of salbutamol syrup. The regulation implication for the originator ciprofloxacin suspension however, resulted in an overall decrease in both the logistics fee and the SEP components.

### Insulin preparations (cold-chain)

The majority of insulins in this study do not have any generics available in South Africa, with the exception of Human Insulin. In South Africa all varieties of insulin are biosynthetic and available only as a single strength, 100 units/ml. They do however differ in their duration of action, preservative content and buffering and retarding additives and forms the premise of their classification in this study [18]. A total of 18 insulin preparations (14 originator and 4 generic medicines) were analysed and sub-dived into 6 sections namely, ultra-fast acting, fast acting, intermediate to long acting, long acting insulin analogues, biphasic, biphasic insulin analogues (see Table 4). The median ex-manufacturers price of the products was ZAR 249.5. All preparations with the exception of Insulin Glargine was priced between the ZAR 100 and ZAR 500 price range, which would subject these items to a logistics fee cap of 6 % plus ZAR 4.

Generics were available for fast acting (1 generic); intermediate to long acting (1 generic) and biphasic (2 generics) insulin analogues. For fast acting and intermediate to long acting insulins, the logistics fees, as well as the percentage logistics fee of the generics were neither the highest nor the lowest from all the other insulins in the group. For biphasic insulins, the generics had the lowest logistics fees. However, only one of these generics had the lowest logistics fee percentage in the group, with the other generic having a logistics fee percentage second to the highest priced originator.

After application of the maximum proposed price cap, a mixed response was observed with half the items (3 of which were generics) seeing an increase in the logistics fee and the remaining nine seeing a decrease in the logistics fee and SEP components.

### Statistical analysis

The t-test was found to be statistically non-significant for all generic and originator medicines for all dosage forms with the exception of generic solid oral dosage forms where *P* = 0.0002 and for generic oral liquid dosage forms where the *t*-test could not be performed owing to the nature of the data.

Table 5 depicts a summary of all the median logistics fee percentages for all four dosage forms surveyed. Results reveal that the logistics percentage of the SEP for originator medicines was higher than that for the generic medicines for injectable and oral liquid dosage forms. The contrary was observed for solid oral dosage forms and insulins.

**Table 5:** Median logistics fee/SEP % for each dosage form based on 2013 prices

Dosage Form	Logistics fee/SEP %
Solid Oral Dosage forms	
Originator	7.8
Generic	7.98
Injectable Dosage Forms	
Originator	8.06
Generic	7.8
Oral Liquid Dosage Forms	
Originator	8.78
Generic	5.04
Insulins	
Originator	5.26
Generic	6.49

### Summary observations for all dosage forms

Overall it was observed that for all medicines surveyed the ex-manufacturer’s price, logistics fee and SEP for originator medicines were higher than those for generic medicines. The only exceptions were observed for the injectable dosage forms, omeprazole and diclofenac, for which the lowest priced generic medicines, had a higher logistics fee than the originator medicine. Furthermore the logistics fees attached to the cold chain medicines were the highest when compared to all the other dosage forms. In summary, majority of the medicines (68 %) surveyed saw an increase in the SEP with the implementation of the proposed tiers for the logistics fee portion of the SEP.

## Discussion

South Africa has embarked on the implementation of several policies to create a transparent pricing system for medicines. The purpose of the proposed maximum logistics fee regulation is to limit pharmaceutical company’s ability to exploit market power by charging high prices to market their specific medicines. In this study we sought to determine the effects of the proposed regulation so as to elucidate potential unintended consequences of the regulation.

Out of the 47 medicines in the overall sample from the current study, only 16 medicines showed a decrease in the SEP with the application of the maximum logistics fee cap. The greatest benefit was observed for medicines where no generic alternative was available. The lack of competition for these medicines may have resulted in pharmaceutical companies setting high prices. The logistics fee cap in these instances has seen a decrease in the SEP for all 3 originator medicines. Thus one could recommend that the logistics fee caps be implemented selectively for those medicines for which there are no generic alternatives currently available.

A medicine price survey conducted by Xiphu and Mpanza in the Gauteng province (2004), South Africa revealed that logistics fees of similar products with similar pack sizes were generally higher for innovator brands than for generics [19]. The results from the current study reveal similar findings for all products surveyed with the exception of only 2 medicines, omeprazole and diclofenac injections. However if one had to look at the logistics fee as a percentage of the final SEP, results reveal that the logistic portion of several of the generic medicines reviewed were higher than that of the originator brand medicine, which could indicate that pharmaceutical companies used their logistics fees to provide a market incentive for wholesalers and distributors to distribute their products along the supply chain. A valid recommendation from the Xiphu, and Mpanza study was the implementation of uniform distribution fees of similar products, with clear stipulation of distribution costs and activities being paid to all stakeholders.

The cold chain which is often considered as a combination of being a science, a technology and a process, has often been cited by pharmaceutical companies as a major contributing factor to the increased prices of certain thermo-labile medicines. These companies suggest that this is due to the difference in logistic planning and assurance of appropriate temperature conditions along the supply chain, which results in a higher cost associated with retaining, packaging and transportation of products at 2–8 ° C or at frozen temperatures than it does to keep them at uncontrolled ambient conditions [20]. In this study, a comparative look at the logistics fees for all dosage forms supports the notion that cold chain medicines do attract higher logistics fees. However when one looks at the current logistics fees component of the SEP of these cold chain products, it becomes apparent that the logistics fee is not the largest determinant of the SEP, especially when compared to other dosage forms (Table 5). Hence the logistics fees associated with maintaining the cold chain is not a major contributor to the high cost of these products as previously suggested. These results however only reflect a small therapeutic class in a larger category of cold-chain products, and conclusions cannot be generalised to the larger majority; however results once again show that the imposition of a logistics fee cap may not be a “fit one, fit all” regulation.

The application of a proposed maximum logistics fee could potentially have an adverse impact on the distribution chain in South Africa, especially since logistics fees attached to medicines may differ, depending on the type or class of medicine. Reduction in overheads and overall business costs is a direct reaction to price cap regulations. Price cap regulations create an incentive for businesses to reduce service quality [21] which would be counterproductive to the objectives of the NDP. As an alternative to instituting price caps, further economic studies into the proposed logistic fee determination should also be looked into. A study conducted in Norway (2009) sought to compare the newly introduced reference price (RP) system called “index pricing” for a sub-sample of off-patent pharmaceuticals to the previous price cap (PC) regulation [22]. The study revealed that RP significantly reduced both brand-name and generic medicine prices within the reference group, with the effect being stronger for brand-names. In terms of regulatory implications, the results suggest that RP is more effective than PC regulation in lowering medicine prices, and perhaps South Africa needs to pursue all options before settling on the intended regulation.

Owing to price variations between different medicines, dosage forms, therapeutic classes and patent status , the proposed logistics fee caps could possibly be implemented under separate strategies for different medicines i.e. selective mark-up regulation [9]. A similar type of selective mark-up regulation has been implemented in India (under the Drugs Price Control Order 2013), where only a select group of medicines based on essential drug classification, are subject to price regulation [23]. The formula adopted includes the regulation of wholesalers and retailers in the maximum retail price that is determined. The end result has been a lower average margin on these selected medicines, thus increasing financial access. Indonesia has developed a system where mark-ups for originator brands were lower than those for generic products (published in 1989 so dated) [9]. South African regulators should explore all avenues of grouping medicines, either via patent status (originator brand, branded generic, generic), innovation status, country of manufacture, reimbursement status, presence on an essential medicines list or other positive lists [9]. This mechanism may not be the definitive answer to reducing costs in the supply chain, as stakeholders may find ways to distort selective mechanisms to their advantage, but may indeed form part of a larger policy objective to curtail costs and ensure transparency in pricing. Alternate methods to reduce pharmaceutical costs may also include strategies to improve the efficiency of the distribution chain. Establishing an appropriate system will not be easy, as it is essential to find an equitable balance between cost effective services and promoting increased accessibility to medicines, without a loss in quality of logistics services.

The European Union (EU) has also recognised how costs in the distribution chain have contributed to escalating costs of medicines. Regulators have introduced changes relating to the remuneration of pharmaceutical wholesalers and pharmacies, with the aim of reducing these costs with an increasing focus on improving the efficiencies of the supply chain [24]. The concern however with the regulation of pharmaceutical prices is that it may distort the behaviour of market players. Regulated margins in EU countries often takes the form of regressive mark-ups, proportional mark-ups and flat fees, each having their own advantages and disadvantages. Regressive mark-ups which have been identified as the most common in the EU for wholesalers are based on a percentage of the ex-manufacturer price that decreases as the price increases. This implies that distributors obtain higher remuneration from supplying higher-price medicines, which in turn would hinder other policies such as generic uptake. Only fixed fees whose value is independent from the medicines prices could in fact fully counterbalance this. The lesson is that any regulation applied early in the supply chain, must be counterbalanced later in the chain with careful consideration of how industry players may manipulate it, to attain the desired outcome.

Some future recommendations stemming from the findings of this preliminary study are firstly to analyse the relationship between logistics fees and the number of generic brands available, to determine if logistics fees plays a role in price competition in the broader South African medicines market. Secondly, further studies are required to determine if high logistics fees are a result of profiteering or whether they do actually reflect high costs in the distribution chain. Thirdly, more investigations are needed to determine if alternative policies such as reference pricing are more effective than price caps on logistics fees in lowering medicine prices. Finally research should be steered toward determining if logistics fee caps have a differential impact on originator brand and generic medicines.

### Limitations

The primary limitation of this study is that, for each medicine surveyed, the maximum logistics fee cap was imposed. Hence for medicines that resulted in a price decrease, the regulation could in reality reflect actual price decreases upon implementation. However, for medicines that resulted in a price increase, this might not be a true reflection of what will necessarily happen, as we cannot predict how manufacturing firms will react or alter prices to remain competitive. Price caps may unfortunately result in perverse economic effects for businesses to recover their lost profits. It therefore becomes imperative for government to strive to increase the transparency in determining the logistics fees which are currently privately negotiated between manufacturers and logistics providers. This possibly could be achieved through logistics fees contracts being public or through negotiations between national Department of Health and wholesalers and distributors. Finally the limited number of oral liquid dosage forms surveyed may affect the generalizability of the results.

## Conclusion

Despite the often good intent of pharmaceutical regulations in trying to reduce medication costs, they may also produce negative overall consequences to the end users. This study reveals that there is indeed a need for greater transparency of the activities being paid for under distribution as well as greater uniformity in the costing of logistics fees of similar products. The implementation of price caps may not necessarily result in reduced prices all round. The results of the study confirm that despite the existence of several avenues to improve the affordability of medicines, each opportunity comes with its own challenges which require suitable lines of response. This trade-off therefore requires reliable and regular monitoring of implemented regulation and policies, together with the allowance for adjustments to ensure that improved desired outcomes are achieved. Any country seeking to emulate the SEP regulation of South Africa, should first ensure that transparency in the determination of the ex-manufacturer price component and in the logistic component be outlined first, before implementing this fixed price policy. They would thus avoid the need to address this non-transparency issue post regulation implementation.

## Abbreviations

EU: European Union
NDP: National Drugs Policy
OECD: Organisation for Economic Co-operation and Development
PC: price cap
RP: reference price
SEP: Single Exit Price
VAT: value added tax
WHO/HAI: World Health Organization and Health Action International
ZAR: South African Rand
